# Supplementary material for: Deciphering Molecular Mechanisms Involved in the Modulation of Human Aquaporins’ Water Permeability by Zinc Cations: A Molecular Dynamics Approach
Source: Int J Mol Sci. 2024 Feb 14;25(4):2267. doi: 10.3390/ijms25042267 (PMC10888569; doi:10.3390/ijms25042267)
Supplement: Supplementary file 1 [file ijms-25-02267-s001.zip › ijms-2850338-supplementary.pdf]

Supplementary

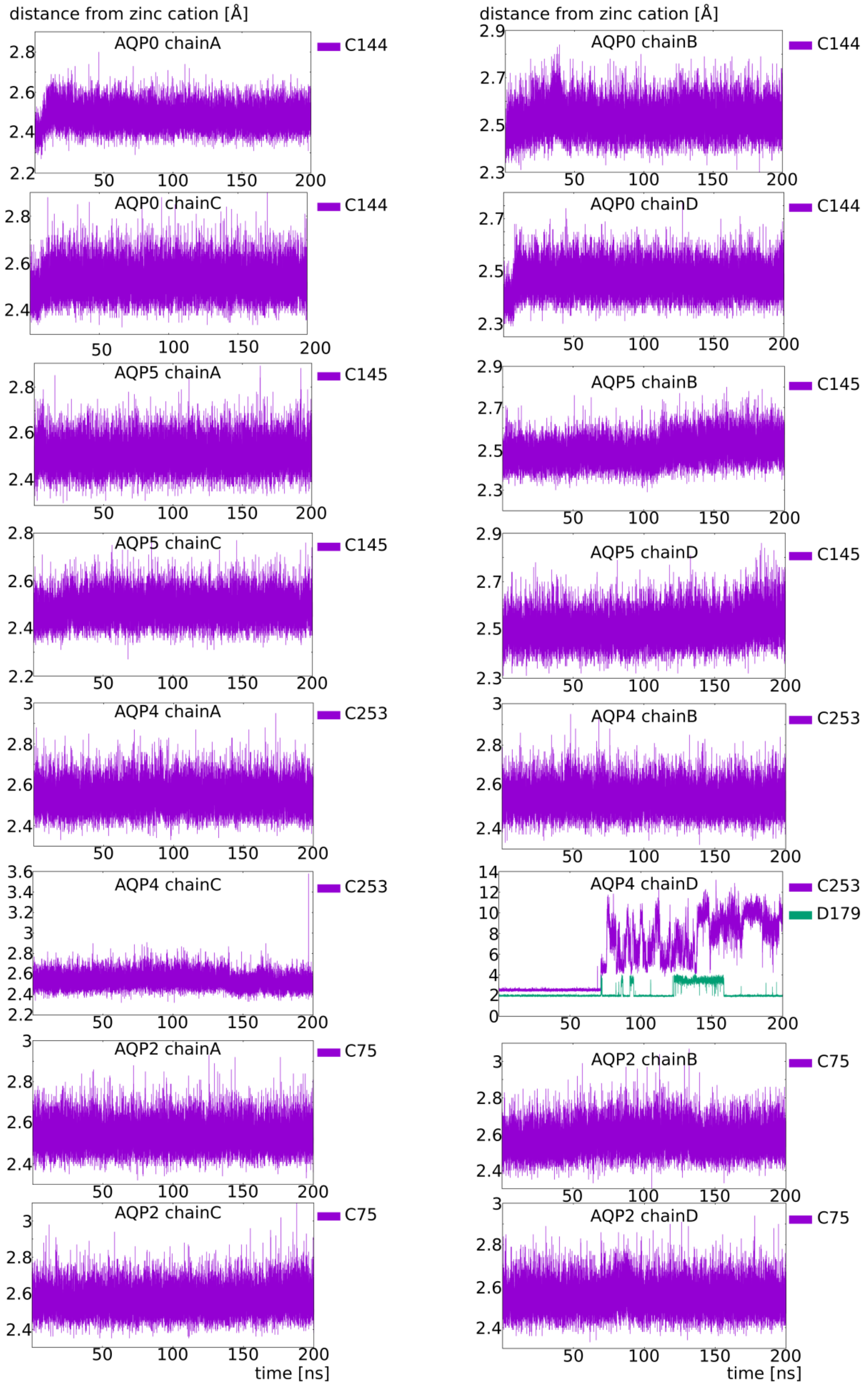


Supplementary figure S1. Zinc cations are continuously bound to their putative binding site. Distances between zinc cations and residues from their putative binding site for each condition

studied are displayed as a function of simulation time. For each condition, cysteine residues were used to compute distances since they are well established as zinc – binding residues. For all conditions, zinc cations are maintained at ~2.5 angströms from the cysteine which is compatible with the formation of a salt bridge. Only for chainD of AQP4 is this link broken. However, another residue of the putative zinc binding site (aspartate 179) is still in interaction with the cation.
